# Reactive Oxygen Species Generation and Use of Antioxidants during *In Vitro* Maturation of Oocytes

**DOI:** 10.22074/ijfs.2017.4995

**Published:** 2017-02-16

**Authors:** Mozafar Khazaei, Faranak Aghaz

**Affiliations:** Fertility and Infertility Research Center, Kermanshah University of Medical Sciences, Kermanshah, Iran

**Keywords:** Oxidative Stress, Reactive Oxygen Species, Antioxidant, *In Vitro* Maturation

## Abstract

*In vitro* maturation (IVM) is emerging as a popular technology at the forefront of fertility treatment and preservation. However, standard *in vitro* culture (IVC) conditions usually increase reactive oxygen species (ROS), which have been implicated as one of the major causes for reduced embryonic development. It is well-known that higher than physiological levels of ROS trigger granulosa cell apoptosis and thereby reduce the transfer of nutrients and survival factors to oocytes, which leads to apoptosis. ROS are neutralized by an elaborate defense system that consists of enzymatic and non-enzymatic antioxidants. The balance between ROS levels and antioxidants within IVM media are important for maintenance of oocytes that develop to the blastocyst stage. The effects of antioxidant supplementation of IVM media have been studied in various mammalian species. Therefore, this article reviews and summarizes the effects of ROS on oocyte quality and the use of antioxidant supplementations for IVM, in addition to its effects on maturation rates and further embryo development.

## Introduction

*In vitro* embryo production (IVEP) allows the production of a high and inexpensive number of embryos to conduct basic research and apply emerging biotechnologies such as cloning and transgenesis. IVEP is a three-step methodology that comprises the following procedures: i. *In vitro* maturation (IVM) of oocytes recovered directly from follicles, ii. In vitro fertilization (IVF) or co-incubation of capacitated spermatozoa with *in vitro* matured oocytes, and iii. *In vitro* culture (IVC) of zygotes up to the blastocyst stage. According to reports, IVM is the key factor that determines the proportion of oocytes which develop to the blastocyst stage.

IVM of oocytes is a complex process influenced by the interplay of regulatory factors that include gonadotrophins and a growing list of secreted molecules, the biochemical state of the oocyte, and interactions between the oocyte and cumulus cells (1- 5). Therefore, the *in vitro* advancement of an oocyte from the diplotene stage of prophase I [germinal vesicle (GV)] to metaphase II (MII), along with cytoplasmic maturation that encloses a broad set of still ill-defined cellular events are essential for fertilization and early development of the embryo (6-8).

Although substantial progress has been made to improve the efficiency of an IVM protocol, however, there is a lack of consistency in the success rate of conventional *in vitro* matured oocytes compared to *in vivo* matured oocytes. Multiple factors likely contribute to the overall poor quality of in vitro matured oocytes. One of the important factors may be oxidative stress (OS). The generation of pro-oxidants such as reactive oxygen species (ROS) is an invariable phenomenon in the culture condition. It is possible that OS also influences oocyte development *in vitro*. On the other hand, ROS are considered signal molecules in oocyte physiology and their impact on maturation promoting factor (MPF) destabilization has recently been reported (9-11).

Oocyte protection against ROS may play important roles in pre-implantation embryonic development. On the other hand, antioxidants are ROS scavengers, thereby helping to maintain the oocyte’s oxidant/antioxidant balance. The effects of antioxidant supplementation to IVM media have been studied in various mammalian species (12-14). Our purpose was to incorporate the role of ROS in oocyte physiology, impact of OS in downfall of oocyte quality (15, 16), and the role of enzymatic as well as non-enzymatic antioxidants in reducing ROS levels and deterioration of oocyte quality under IVC conditions. This review article summarized the effects of ROS, the use of antioxidant supplementations for IVM, and its effects on maturation rates. In this systematic review, we used IVM, OS, ROS, and antioxidant as keywords from scientific databases between 1990 and 2016. After a review of all abstracts, we included strong, reliable research in this report.

### Production of reactive oxygen species and generation of oxidative stress

OS is caused by an imbalance between pro-oxidants and antioxidants (17). This ratio could change with increased levels of pro-oxidants, such as ROS, or a decrease in antioxidant defense mechanisms (18-20). ROS represents a wide class of molecules that indicate the collection of free radicals (hydroxyl ion, superoxide, etc.), non-radicals (ozone, single oxygen, lipid peroxides, hydrogen peroxide) and oxygen derivatives (21). They are highly reactive and unstable. Hence, ROS can react with nucleic acids, lipids, proteins, and carbohydrates to acquire an electron and become stable. These reactions induce a cascade of subsequent chain reactions that eventually result in cell damage (22-24). ROS can diffuse and pass through cell membranes and alter most types of cellular molecules (nucleic acids, proteins, and lipids), leading to mitochondrial alterations (25), meiotic arrest in the oocytes (26), embryonic block, and cell death (27). On the other hand, OS occurs when increased ROS levels which disrupt cellular redox circuits, result in disturbances of redox-regulated cellular processes and/or oxidatively damage cellular macromolecules (28).

### Oxidative stress and in vitro maturation

Under physiological conditions, the oocytes are major sources of ROS because they use oxygen to produce energy through mitochondrial oxidative phosphorylation. Their ROS production is increased during IVM when compared to *in vitro* maturation (13, 29). Increased levels of ROS beyond the physiological range that may lead to OS can result in deterioration in oocyte quality and thereby affect reproductive outcomes (30). A better understanding of the OS status and its regulation during IVM is needed. However, one must also consider whether and how OS may influence the process of IVM. This section focuses on reports that refer to mechanistic roles for OS in oocyte maturation, especially with respect to key features of nuclear and cytoplasmic events within the oocyte.

### Reactive oxygen species and nuclear and cytoplasmic maturation

**Fig.1:**
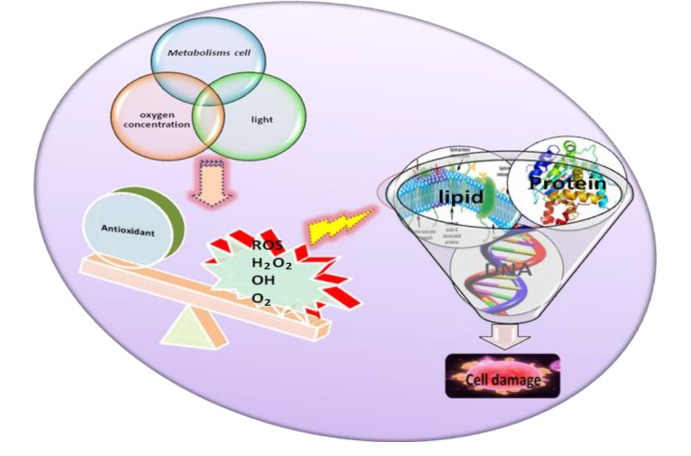
The possible factors that induce generation of reactive oxygen species (ROS) in the oocyte. The imbalance between ROS and antioxidants, the impact of high levels of ROS, and the resulting oxidative stress (OS) on meiotic arrest and apoptosis in oocytes.

Increased levels of ROS associated with induce cell cycle arrest in human oocytes as well as in mouse embryos (31). A multitude of key factors regulate the generation of ROS in the media and include various cellular metabolic reactions, oxygen concentration, light, oocyte handling, and general physicochemical parameters that may have a negative impact on oocyte physiology by inducing apoptosis (Fig .1). One of the major constituent that may alter developmental responses in the oocyte is relevant to OS since light is known to result in an imbalance of pro- and antioxidants in somatic cells and embryos. Similarly, a relationship has been shown in a mouse model between a type of light commonly used in the laboratory with increased ROS concentrations and compromised embryonic and fetal development (32). Oxygen tension is another important difference between the *in vivo* and *in vitro* environments for the oocyte culture. Toxic effects of atmospheric oxygen concentration under standard culture conditions and the beneficial effects of lower O_2_ concentrations (5-7%) on developmental competence of oocytes *in vitro* have been reported in mice (33, 34), hamsters (35, 36), rats (37), sheep and cattle (38-40), and humans (41-43).

The conditions of an IVC generate ROS, which could exert some beneficial effects if the ROS levels remain under physiological levels (44). The tonic generation of ROS triggers meiotic resumption from diplotene as well as the MII arrest stage in several mammalian species (44, 45). It has been reported that levels of ROS beyond the physiological range could induce destabilization of maturation MPF, reduce survival factors, and trigger mitochondria-mediated apoptosis of oocytes (15, 46). The biphasic role of ROS must be sufficiently discussed in order to update OS and its impact on oocyte quality (15). The beneficial role of ROS comes from the observations that non-enzymatic antioxidants, such as ascorbic acid and 3-tert-butyl-4-hydroxyanisole (BHA), inhibit spontaneous meiotic resumption from diplotene arrest (47). These results suggest a beneficial threshold level for ROS.

### Antioxidants

Antioxidants scavenge ROS, which helps maintain the cell oxidant/antioxidant balance. On the other hand, antioxidants are the compounds which either suppress the formation of ROS or oppose their actions. There are two types of antioxidants: enzymatic and non-enzymatic (Table 1).

**Table 1:** List of studies that show the effects of antioxidant supplements that improve *in vitro* maturation


Antioxidant	Experimental model

Enzymatic antioxidants	
Superoxide dismutase (SOD)	Mouse
Thioredoxin	Porcine
Catalase (CAT)	Bovine
Sericin	Bovine
Non-enzymatic antioxidants	
Glutathione (GSH)	Hamster, pig, ovine,
	Bovine and equine
Cysteamine	Canine, mice, goats, porcine
Vitamin C (Ascorbic acid)	Mouse
Vitamin E and trolox	-


**Fig.2:**
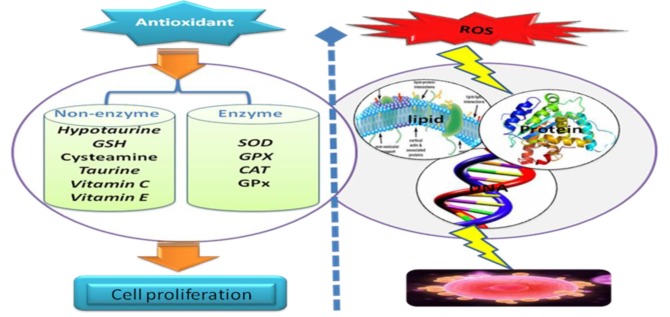
The presence of antioxidant enzymes such as superoxide dismutase (SOD), glutathione peroxidases (GPx), and catalase (CAT) as well as non-enzymatic antioxidants, such as vitamin E and C (ascorbic acid), glutathione (GSH), uric acid, and albumin in the oocytes. Excess amounts of reactive oxygen species (ROS) may be involved in oxidative stress (OS) of oocytes and granulosa cells.

Enzymatic antioxidants neutralize excess ROS and prevent it from damaging the cellular structure. Enzymatic antioxidants are composed of superoxide dismutase (SOD), catalase (CAT), various peroxidases and peroxiredoxins (PRDXs), including glutathione peroxidases (GPXs), which can convert peroxides to water and alcohol (48). SOD enzymes catalyze the dismutation of superoxide anion (O_2_^-^) into O_2_ and HO_2_O_2_ while CAT converts HO_2_O_2_ to O_2_ and H2O. The enzyme SOD exists as three isoenzymes (49): SOD1, SOD2, and SOD3. SOD1 contains Cu and zinc (Zn) as metal co-factors in the cytosol. SOD2 is a mitochondrial isoform that contains manganese (Mn), whereas SOD3 encodes the extracellular form. Nutrients such as Se, Cu, and Zn are required for the activities of some antioxidant enzymes, although they have no antioxidant actions. Non-enzymatic antioxidants are composed of glutathione (GSH), vitamin C, taurine, hypotaurine, vitamin E, Zn, selenium (Se), beta carotene, and carotene (47). GSH is a tripeptide thiol compound with many important functions in intracellular physiology and metabolism. One of the most important roles of GSH is to maintain the redox state in cells which protects them against harmful effects effects caused by oxidative injuries. The protective action of GSH against ROS is facilitated by the interactions with its associated enzymes, such as GPx and GSH reductase (Fig .2).

Vitamin C (ascorbic acid) is a known redox catalyst that can reduce and neutralize ROS (50). Based on its chemical structure, ascorbic acid is an electron donor and therefore a reducing agent. It has two different biochemical roles-antioxidant and enzymatic cofactor. Ascorbic acid is maintained through reactions with GSH and can be catalyzed by protein disulfide isomerase and glutaredoxins. Cysteamine is a low-molecular weight amino acid that contains thiol (51). The addition of cysteamine not only enhances the GSH content in MII oocytes but also protects the membrane lipids and proteins due to indirect radical scavenging properties (52). The concentrations of many amino acids, including taurine and hypotaurine are non-enzymatic antioxidants that help maintain the redox status in oocytes (53).

Vitamin E (α-tocopherol) is a lipid soluble vitamin with antioxidant activity. It consists of eight tocopherols and tocotrienols. Vitamin E may directly destroy free radicals such as peroxyl and alkoxyl (ROO•) generated during ferrous ascorbate-induced lipid peroxidation (LPO), thus it is suggested as a major chain breaking antioxidant (54). Hyaluronan, melatonin, tea and sericin are known to act as indispensable antioxidants in IVEP. They can block the release of pro-oxidant factors released as a result of OS (12, 55, 56).

Hyaluronan, an essential component of the extracellular matrix and non-sulfated glycosaminoglycan may play an important role in meiotic resumption of oocytes (57). The hormone melatonin (N-acetyl-5-metoxy tryptamine) is an antioxidant that, unlike GSH and vitamins C and E, is produced by mammals. In contrast to other antioxidants, however, melatonin cannot undergo redox cycling. Once oxidized, it is unable to return to its reduced state because of the formation of stable end-products after the reaction (14). As an antioxidant, green tea has been shown to improve IVM and embryo development of sheep COCs to the blastocyst stage in IVM medium (58). Sericin a water-soluble globular protein (protein hydrolysate) is derived from the silkworm Bombyx mori. This protein represents a family of proteins whose molecular mass ranges from 10 to 310 kDa (59). Dash et al. (60) have reported that sericin might provide a protective effect on fibroblasts by promoting endogenous antioxidant enzymes *in vitro*.

### Antioxidant supplements and improving in vitro maturation

The addition of enzymatic antioxidants such as SOD, CAT, and thioredoxin are effective for pre-embryo development as scavengers of ROS and serving embryos a low OS condition in mice (61, 62), porcine (63), and bovines (64). Sericin, an antioxidant protein, improves embryo development (60, 65) and is a critical supplement for oocyte maturation (12, 56).

A series of non-enzymatic antioxidants protect oocytes against ROS damage during oocyte maturation. GSH is one of the naturally synthesized antioxidants that protect cells from ROS toxicity and regulate the intracellular redox balance (66). The intracellular level of GSH increases during oocyte maturation in hamsters (67), pigs (68), ovine (69), bovines (70), and equines (71). Recent reports have shown that addition of low molecular weight thiol compounds, such as cysteamine and b-mercaptoethanol to IVM media improved the cytoplasmic maturation of oocytes and embryo development by increasing GSH synthesis (66, 72, 73).

Cysteamine supplementation during IVM reportedly improved nuclear maturation rates in canines (74), mice (75), goats (76), and porcine (77). Although, other studies in goats (78), pigs (79), horses (13), buffalos (80), and cattle (81) did not show any increase in nuclear maturation rates. Addition of cysteamine to the IVM medium improved embryo development to the blastocyst stage in mammalian oocytes (82).

Ascorbate is concentrated in granulosa cells, theca cells, luteal cells, and oocytes (28). Choi et al. (83) reported a beneficial role for vitamin C in protecting spindle structures of MII mouse oocytes and chromosomal alignment against an oxidant (hydrogen peroxide)-induced damage. It is suggested that the effect of vitamin C is associated mainly with its capability to promote ooplasmic maturation during IVM. The beneficial role of ROS comes from the observations that non-enzymatic antioxidants, such as ascorbic acid, inhibit spontaneous meiotic resumption from diplotene arrest. We have presented a number of these observations. Tatemoto et al. (84), Kere et al. (85), and Córdova el al. (86) found that the addition of vitamin C to the oocyte maturation medium exerted no effect on the maturation rates of oocytes. Similarly, antioxidants such as vitamin E and trolox had no effect on oocyte maturation, but other antioxidants such as propyl gallate and 2,4,5-trihydroxybutrophenone inhibited the spontaneous resumption of meiosis (87). Together, these studies emphasized the beneficial roles of ROS during IVM at certain concentrations (low level).

## Conclusion

It is well-known that high levels of ROS beyond the physiological range could induce MPF destabilization, reduce survival factors, and trigger apoptosis in oocytes of several mammalian species. Antioxidants are the main defense factors against OS induced by ROS. Many reports suggest that antioxidant supplementation of IVM media improves cytoplasmic maturation by alleviating OS during oocyte maturation via increasing GSH storage, and contributes to further protect the embryo against oxidative aggressions during its early developmental stages. On the other hand, supplementation by antioxidants during IVC improves oocyte quality by reducing ROS levels and apoptotic factors. However, some of the non-antioxidants such as ascorbic acid and 2, 4, 5-trihydroxybutrophenone do not improve oocyte maturation; rather, they inhibit spontaneous resumption of meiosis. Improvements to culture conditions are complex challenges that depend not only on the choice of an antioxidant but also on its concentration, the medium and its components, the species, and the dynamic changes of the specific requirements of the oocyte according to its developmental stage. Future efforts should be placed on understanding the involvement of ROS in oocyte apoptosis and for guiding antioxidant-based strategies to selectively control ROS-induced damage without compromising the physiological functions of these species.
